# Editorial: MiRNAs as pivotal components of ncRNA networks associated with CNS injuries and neurodegeneration, and their therapeutic potential

**DOI:** 10.3389/fnmol.2023.1166943

**Published:** 2023-03-13

**Authors:** Danae Campos-Melo, Cristian A. Droppelmann, Ling Q. Zhu

**Affiliations:** ^1^Molecular Medicine Group, Robarts Research Institute, Western University, London, ON, Canada; ^2^Huazhong University of Science and Technology, Wuhan, China

**Keywords:** miRNA, ncRNAs (non-coding RNAs), neurodegeneration, spinal cord injury, lncRNAs (long non-coding RNAs), circRNAs

Since miRNA discovery 30 years ago, studies have been focused on understanding miRNA mechanisms of action and biogenesis, as well as the pathways they regulate and their potential role in diagnosis and therapy of different diseases (Dexheimer and Cochella, 2020; Diener et al., 2022; Matulic et al., 2022; Rani and Sengar, 2022). Many studies have revealed alterations in the expression of miRNAs under different physiological and pathological conditions, supporting their participation in all cellular processes (Deveale et al., 2021). Evidence shows that miRNAs respond to different environmental stimuli, such as cellular stress and viral infections, regulating gene expression in different cell types and organisms (Gebert and Macrae, 2019; Deveale et al., 2021). The high stability and rapid expression of miRNAs compared to other RNA species make them ideal candidates in charge of the fine-tuning of mRNA and protein levels, and ultimately of the cellular response to diverse clues (Campos-Melo et al., 2022). These characteristics and the regulatory networks that miRNAs establish interacting with other non-coding RNAs (ncRNAs) such as long non-coding RNAs (lncRNAs) and circular RNAs (circRNAs), are critical for neuronal function, death, and survival and are increasingly recognized as associated with human neurological disorders, such as neurodegeneration, spinal cord injury, and stroke (Yamamura et al., 2018; Nuzziello and Liguori, 2019; Xu et al., 2021; Khan et al., 2022; Li et al., 2022; Silvestro and Mazzon, 2022).

This Research Topic clusters five articles from experts interested in *in silico* and *in vitro* miRNA characterization, miRNA functional interactions with other ncRNA molecules, and potential therapeutic roles of miRNAs in neurological diseases. Mégret et al. reviewed the main families of machine learning methods for the analysis of miRNA regulation in neurodegenerative disorders. The authors argue that the challenge in system-level studies to analyze the role of miRNA regulation in neurodegenerative diseases is due to problems of insufficient and inhomogeneous data and in the accuracy of system-level modeling. The authors propose shape analysis of complex omics data as a promising approach to construct improved models of high-level precision in matching miRNA-mRNA profiles in neurodegeneration.

Liang et al. reviewed mesenchymal stem cells (MSC)-derived exosomal miRNAs and its potential applications as therapeutic tools. This group of miRNAs downregulates the expression of several genes such as *IRAK1, TRAF6, C/EBP*β *IRF5, TLR4*, and *MAPK6*, which induces polarization of macrophages from M1 to M2 phenotype and promotes nerve function recovery. These miRNAs are proposed to provide better therapeutic opportunities for spinal cord injury (SCI) than MSC transplantation.

Thousands of miRNA sequences hitherto have been published in miRbase (Plotnikova et al., 2019) (https://www.mirbase.org/), however, the big majority of them still require experimental validation. In this topic, two novel miRNAs encoded in the urokinase receptor gene (uPAR) gene *Plaur* which is associated with nerve regeneration, were identified and characterized by Rysenkova et al.. MiRNAs Plaur-miR1-3p and Plaur-miR1-5p, were described to be expressed in mouse brains and target *Mef2D*, a gene that encodes a protein that regulates gene expression in embryogenesis and brain architecture maintenance, and contributes to the regulation of neuronal apoptosis, neurogenesis, and differentiation.

In recent years, several articles have studied regulatory networks and interactions between miRNAs and other ncRNAs *in vitro* and *in vivo*. Both, lncRNAs and circRNAs function as miRNA sponges or decoys altering the availability of miRNAs and their downstream regulatory effects (Liu and Chen, 2022; Sharma et al., 2023; Tang et al., 2023). For this Research Topic, Lan et al. reviewed lncRNAs that are involved in Alzheimer's disease (AD). LncRNAs modulate the transcription of target genes in *cis* or in *trans*, and mRNA stability and processing, by regulating the assembly of multi-molecular complexes. This article reviews evidence that lncRNAs contribute to the pathogenesis of AD by regulating tau hyperphosphorylation, Aβ plague formation, mitochondrial and synaptic function, neuroinflammation, and neuronal apoptosis.

Finally, Zhang et al. extensively reviewed the role of circRNAs, covalently closed ncRNAs, in acute central nervous system (CNS) injuries, such as traumatic brain injury (TBI) and SCI. The authors described circRNAs expressed in neuronal injury as beneficial or detrimental for neural cell function and survival. Details of the function of different circRNAs involved in cerebellar ataxia, ischemic stroke, subarachnoid hemorrhage, and brain injury are included, showing evidence of the neuroprotective function of circRNAs through inhibition of inflammation and suppression of apoptosis, promotion of angiogenesis, and reduction of excitotoxicity and oxidative stress, among others. Mechanisms of action of circRNAs in acute CNS injuries are discussed, including their role in regulating miRNA performance.

From viruses to humans, the regulatory role of miRNAs and their wiring networks with other ncRNAs is only beginning to be elucidated. Beyond the experimental characterization of most miRNAs, we need to understand where, when, and how miRNAs exert their actions over specific targets in the cell. This is particularly relevant in animals because the partial base pairing between the miRNA seed region and 3′UTR sequences creates a high repertoire of targets. Studies of spatiotemporal expression and function of miRNAs and their ncRNA partners within subcellular compartments, in different tissue cell types, and ultimately in the whole organism, will allow the understanding of the importance of genome regulation by miRNAs and their link to human diseases of the CNS.

## Author contributions

DC-M conceived and wrote the manuscript. CD and LZ reviewed and approved the final article. All authors contributed to the article and approved the submitted version.
